# Chyloperitoneum due to gastric carcinoma: a case report

**DOI:** 10.1093/omcr/omac062

**Published:** 2022-06-23

**Authors:** J C Pinto, S Oliveira, L Duarte, M Ferreira, C Marques, C Casimiro

**Affiliations:** Serviço de Cirurgia Geral, Centro Hospitalar Tondela Viseu EPE, Viseu, Portugal; Serviço de Cirurgia Geral, Centro Hospitalar Tondela Viseu EPE, Viseu, Portugal; Serviço de Cirurgia Geral, Centro Hospitalar Tondela Viseu EPE, Viseu, Portugal; Serviço de Cirurgia Geral, Centro Hospitalar Tondela Viseu EPE, Viseu, Portugal; Serviço de Cirurgia Geral, Centro Hospitalar Tondela Viseu EPE, Viseu, Portugal; Serviço de Cirurgia Geral, Centro Hospitalar Tondela Viseu EPE, Viseu, Portugal

## Abstract

Chyloperitoneum is a rare manifestation of gastric carcinoma, generally occurring late in the course of the disease with a poor prognosis. We report an unusual case of chyloperitoneum in a patient with gastric carcinoma. A 61-year-old male patient presented with postprandial fullness, nausea and weight loss. The upper gastrointestinal endoscopy demonstrated a stenosing lesion of the esophagogastric junction. A biopsy was made and revealed a signet-ring cell gastric adenocarcinoma. The staging CT scan showed multiple abdominal lymphadenopathies and mild ascites. The patient underwent a staging laparoscopy that revealed a large carcinoma of the gastric cardia and a milky-appearing peritoneal fluid. A peritoneal washing and abdominal drainage were performed. The fluid analysis showed a high concentration of triglycerides, compatible with a chyloperitoneum. The patient started medium chain triglycerides-based diet with good response. This case report emphasizes that chyloperitoneum should be considered when assessing patients with gastric carcinoma.

## INTRODUCTION

Chyloperitoneum or chylous ascites is a rare form of ascites that results from the spillage of lymph into the peritoneal cavity [1, 2]. It is related to damage or obstruction to the lymphatic system resulting in ascites with a turbid or milky appearance due to the high triglyceride content [3]. Solid organ malignancies are a rare cause of chyloperitoneum with the incidence being <1% [4]. Due to the loss of nutrients and immunoglobulins, it can lead to dehydration, malnutrition and compromised immunity. The 1-year mortality rate of chyloperitoneum due to malignance progression is reported to be 90% so its rapid recognition and treatment are crucial for a better prognosis [4, 5]. Although this complication arises during or after gastrectomy, we report a remarkable case of chyloperitoneum as the presentation of a signet cell-ring gastric carcinoma highlighting the pathogenesis, evaluation and management of this entity.

**Figure 1:**
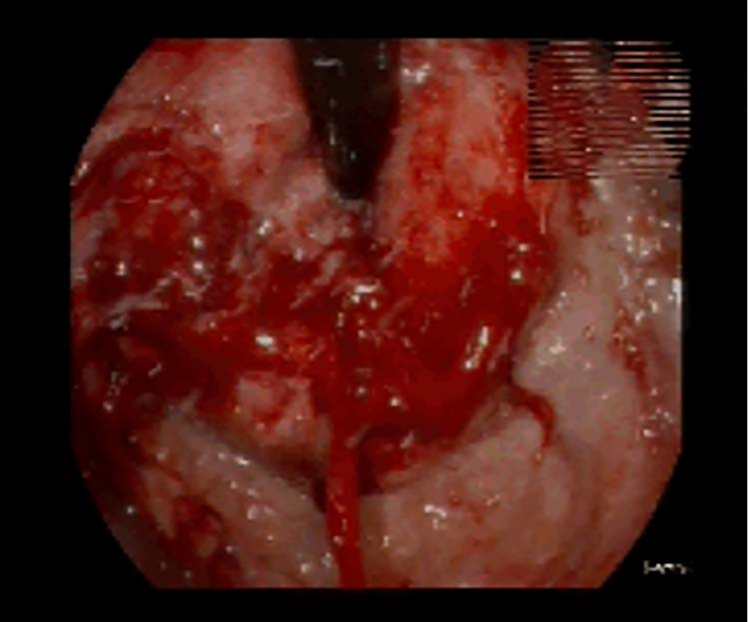
Endoscopic appearance of the tumor.

## CASE REPORT

**Figure 2:**
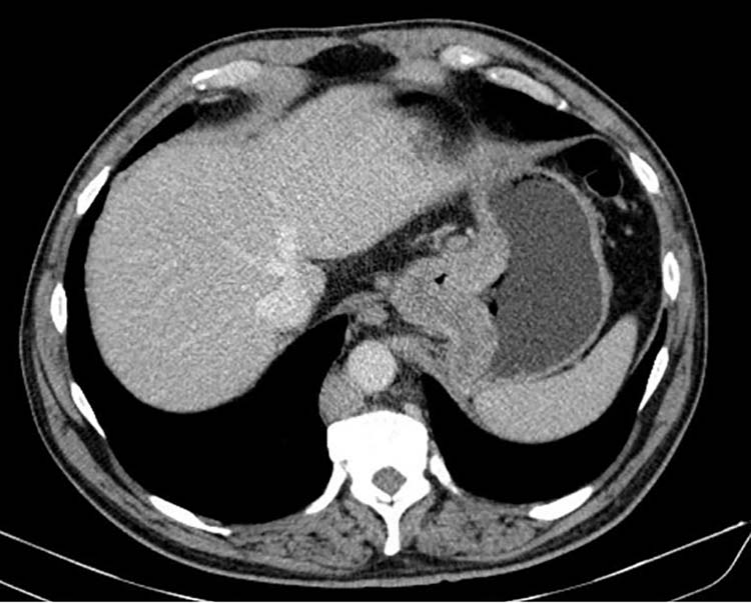
CT scan showing exuberant parietal thickening of the cardia.

**Figure 3:**
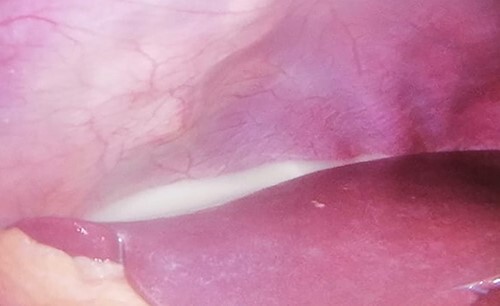
Milky-appearing peritoneal fluid on staging laparoscopy.

A 61-year-old male patient, without any relevant medical record, presented with postprandial fullness, nausea, vomiting and weight loss. The physical examination was unremarkable. The upper gastrointestinal endoscopy demonstrated a circumferential, extensive and friable tumor of the esophagogastric junction and cardia. (Fig. 1) Considering the obstructive nature of this lesion, an endoscopic ultrasound was not possible and dilatation was performed. The biopsy revealed a signet-ring cell gastric adenocarcinoma. The HER2 and PD-L1 status were negative and no microsatellite instability was found. Laboratory workup showed anemia, and elevated tumor markers (CA 19–975.8 U/dL; CA 125–241.8 U/dL). The staging CT scan showed mild ascites without metastatic lesions. (Fig. 2) The patient underwent a staging laparoscopy that revealed a tumor of the gastric cardia with invasion of the diaphragmatic left crus, multiple peritoneal metastatic implants and a milky-appearing peritoneal fluid (Fig. 3) that was collected for biochemical and cytological analysis.

**Figure 4:**
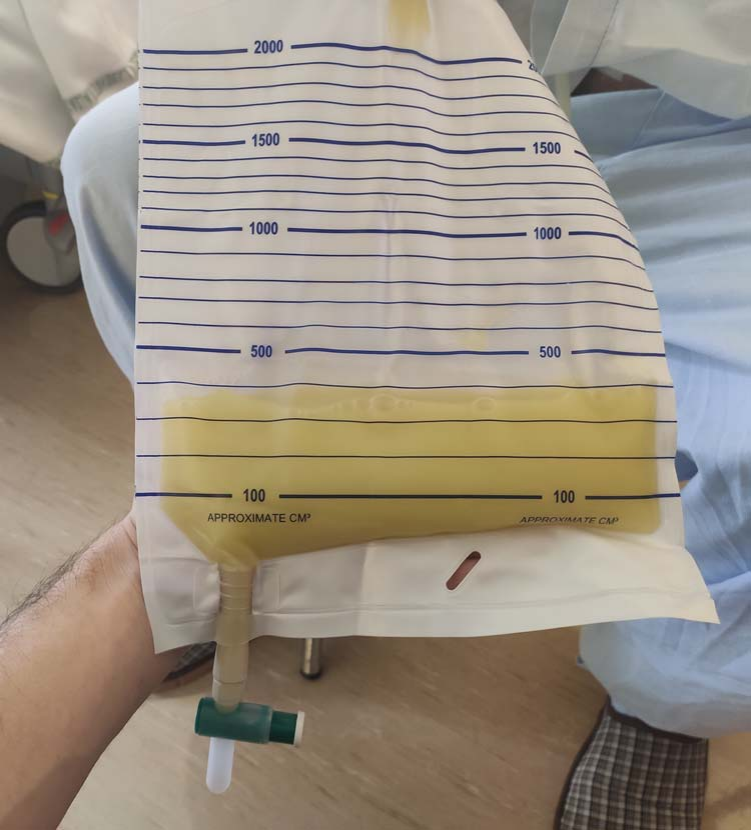
Chylous ascites on abdominal drainage.

A peritoneal washing, abdominal drainage (Fig. 4) and endoscopic placing of a self-expanding metal stent to palliate the obstruction were performed without complications. The fluid analysis confirmed a lymphatic content (pH 7.46, triglycerides 348 mg/dL, total cholesterol 75 mg/dL, amylase 27 UI/L, lymphocyte 0.3%, leucocytes 14.5 x 10^3^), compatible with a chyloperitoneum, and a positive cytology for tumor cells. The patient started a personalized diet program: liquid, low-fat diet enriched with medium chain triglyceride (MCT) oil for the first 3 days after surgery and then a low-fat, normoprotein, pasty diet, with only MCT oil as the added source of fat at the four main meals (Table 1). The liquid drainage volume has progressively decreased and patient experienced an improvement of prealbumin levels and increment of body mass index. Based on these findings, the case was presented in the Cancer Multidisciplinary Team Meeting and chemotherapy, with palliative intent, was proposed. The patient underwent a chemotherapy regimen with FOLFOX (5-FU, oxaliplatin and folinic acid), but the clinical condition worsened and the patient died after 4 months.

**Table 1:** Nutritional information of the diet regimen

	TOTAL CALORIC INTAKE (Kcal)	PROTEINS (g)	CARBOHYDRATES (g)	LIPIDS (g)
DAY 1 TO 4	504	24 (19%)	66 (52%)	16 (29%)
DAY 4 TO DISCHARGED	1514	96 (18%)	195 (52%)	50 (30%)

## DISCUSSION

Chyloperitoneum or chylous ascites refers to the accumulation of lymph into the peritoneal cavity. The lymphatic system is a drainage system that includes lymph, lymphatic vessels and lymphatic tissues that allows the absorption of interstitial fluid, rich in triglycerides and proteins, to the vascular system. Any mechanism that leads to an obstruction or disruption of these channels can lead to the accumulation of lipid-rich lymph [2]. Malignancy is the main cause of chyloperitoneum in western countries and lymphoma accounts for the majority of these cases [2]. Solid organs neoplasms, particularly signet-cell ring gastric carcinoma, are a rare cause of this entity with only a few cases reported [1, 2]. There are several other causes such as cirrhosis or heart failure [3]. There is also one case described of chyloperitoneum secondary to the chemotherapy regimen used to treat this type of carcinomas, which seems to be more frequent during the use of Nivolumab and other immunomodulators [5]. The evaluation and diagnosis should include a detailed history contemplating family history, previous trauma or surgery and medical history, especially malignancy [4]. Chyloperitoneum usually presents with progressive abdominal distension without abdominal pain. Non-specific symptoms as nauseas, anorexia, edema and adenopathy may also be present [4]. Physical examination should be performed and ascites, abdominal masses, edema and sign of malnutrition are common findings. Abdominal paracentesis is the most important diagnostic tool for ascites investigation and can also be performed with therapeutic/palliative intent [2]. In our case, paracentesis was not performed due to the small volume of ascites and the consequent high risk of associated iatrogenesis. Chylous ascites is associated with multiple complications, as nutritional deficiencies increased susceptibility to infection and modified absorption and metabolism of several drugs predicting a poor prognosis [2]. Chyloperitoneum presents as a milky and thick fluid and a triglyceride level superior to 200 mg/dL are the best parameters for establishing its diagnosis [4]. The analysis of the ascitic fluid should also include gross examination, cell count, gram stain, culture, total protein, albumin, glucose, amylase, lactate dehydrogenase and cytology [2, 3]. The serum-ascites albumin gradient should be performed to exclude ascites secondary to portal hypertension as well [3]. Computed tomography, lymphoscintigraphy and lymphangiography are useful imaging studies to identify intra-abdominal lymph nodes, masses, fistulas, leakage or lymphatic obstruction [3]. These techniques are particularly useful to select patients for surgery and to assess the effects of treatment [4]. There is a lack of consensus concerning the best management options for chyloperitoneum, although it is crucial to treat the underlying cause. The cornerstone of therapy is to optimize the patient’s nutritional status and conservative management should be preferred [4]. Dietary therapy based in a high-protein and low-fat diet with MCT that are directly absorbed, reducing the production of chyle, should be the first therapy for chyloperitoneum [3, 4, 6]. The most recent literature shows that in severe cases enteral nutrition with MCT is safer and should be the first option when compared with total parenteral nutrition [6]. Pharmacological treatment with orlistat, somatostatin, octreotide or etilefrine can be attempted with variable results [3]. Paracentesis may provide temporary relief but its frequent use should be avoided because it can result in electrolyte disturbances and infection [4]. Lymphangiography along with percutaneous embolization is a promising minimally invasive technique [7, 8]. Surgical treatment should be reserved to persistent chylous leak or serious nutritional deficits despite optimal dietary and medical management. The volume of chyle drainage should also be taken into account since a drainage over 500 ml a day is a strong predictor of conservative therapy failure [9]. Once the leak is identified, it can be sutured or ligated [8]. The intraoperative use of fibrin glue spraying is a complementary method to prevent recurrent leaking after surgery or when the intraoperative identification of the leak is not possible, a very frequent issue [2, 10]. Regardless the specific therapeutic treatment planned, it is critical to provide adequate nutritional and metabolic support. In our case, considering the low volume of fluid that was being drained, we decided to perform a MCT-based diet, which lead to a progressive decrease of the drainage amount and to the improvement of the nutritional parameters.

For conclusion, chyloperitoneum secondary to a signet-cell ring gastric carcinoma is a rare finding with a wide range of therapeutic options depending on the duration and volume of the leak and the nutritional status of the patient. This case shows that although chyloperitoneum associated with gastric neoplasms is a sign of poor prognosis, conservative therapy based on dietary care is crucial for an improvement in short-term survival and should always be the first step in its approach allowing an improvement in quality of life.

## CONFLICT OF INTEREST STATEMENT

None declared.

## ETHICAL APPROVAL

No ethical approval required.

## CONSENT

Informed consent was obtained from the patient.

## GUARANTOR

José Pinto is the guarantor.
